# Adult-Onset Alexander Disease: A Case Report and Literature Review of Glu207 Alterations

**DOI:** 10.7759/cureus.85617

**Published:** 2025-06-09

**Authors:** Sai Krishna Vallamchetla, Omar Abdelkader, Ibrahim S Tuna, Gayane Barsamyan, Hans Shuhaiber

**Affiliations:** 1 Department of Neurology, Mayo Clinic, Jacksonville, USA; 2 Department of Radiology, University of Florida College of Medicine, Gainesville, USA; 3 Department of Medicine, University of Florida College of Medicine, Gainesville, USA; 4 Department of Neurology, University of Florida College of Medicine, Gainesville, USA

**Keywords:** alexander, alexander disease, autosomal dominant disorder, gfap, leukodystrophy

## Abstract

Alexander disease (AxD) is a rare leukodystrophy caused by heterozygous mutations in *GFAP*. We report the case of a 67-year-old woman with progressive dysarthria, dysphagia, ataxia, and oculomotor dysfunction. MRI revealed medullary and cervical spinal cord atrophy with a periventricular T2/fluid-attenuated inversion recovery (FLAIR) hyperintense rim, findings characteristic of adult-onset AxD. Genetic testing identified a heterozygous *GFAP* c.620A>T (p.Glu207Val) variant, absent from major population databases. Her symptomatic brother carried the same mutation, and additional maternal relatives exhibited suggestive neurological features, supporting autosomal dominant inheritance with variable expressivity. In silico tools predicted the variant to be pathogenic, and multiple mutations at the same residue have been associated with AxD. This case expands the genotypic spectrum of adult-onset AxD, reinforces the diagnostic value of characteristic imaging findings, and underscores the importance of considering *GFAP* testing in adults with unexplained bulbar and pyramidal signs. Early recognition facilitates targeted symptomatic management and informs genetic counseling in affected families.

## Introduction

Alexander disease (AxD) is a rare, fatal neurodegenerative leukodystrophy caused by heterozygous gain-of-function mutations in the *GFAP* gene (chromosome 17q21) encoding glial fibrillary acidic protein (GFAP), an astrocyte-specific type III intermediate filament [1]. It is characterized pathologically by the abnormal accumulation of Rosenthal fibers in astrocytes, which are ubiquitinated protein aggregates composed of GFAP, heat shock protein 27 (HSP27), and alpha B-crystallin [2]. Due to variability in phenotypic expression and its strong correlation with age of onset, AxD was grouped into two major subtypes. Type I (early onset, often < 4 years) is characterized by macrocephaly, seizures, developmental delay, and failure to thrive. In contrast, Type II (older children and adults) is characterized by a slowly progressive course with bulbar, cerebellar, and oculomotor signs with autonomic dysfunction. These subtypes exhibit distinct neuroimaging patterns. Infantile forms typically show extensive frontal white matter abnormalities, basal ganglia involvement, and a periventricular rim, whereas the adult form often presents with mild-to-moderate cerebral involvement, atrophy of the medulla and cervical spinal cord, and signal abnormalities in the anterior portion of the medulla oblongata [3].

Diagnosis of AxD is suspected based on clinical presentation with supportive neuroimaging findings and is confirmed by identifying pathogenic *GFAP* mutations on genetic testing [4]. At least 182 distinct* GFAP *mutations had been identified in individuals with AxD, with the majority (97.4%) being missense mutations [1]. However, the phenotypic spectrum and underlying genetic landscape of leukodystrophies remain complex, with many mutations yet to be fully characterized [5]. Identifying novel mutations is crucial for improving diagnostic accuracy, understanding genotype-phenotype correlations, and guiding genetic counseling.

Here, we describe the case of a 67-year-old woman who presented with progressive bulbar dysfunction, spastic-ataxic gait, oculomotor abnormalities, and a suggestive family history, and was found to have a rare heterozygous missense mutation in the *GFAP* gene, specifically the c.620A>T (p.Glu207Val) variant. This case contributes to the evolving genotypic spectrum of adult-onset AxD and highlights the importance of considering *GFAP* testing in adults with unexplained bulbar-pyramidal syndromes.

## Case presentation

A 67-year-old woman presented to our clinic with a two-year history of progressive dysarthria, dysphagia, worsening balance, and gait instability. She also reported intermittent episodes of dizziness, chronic constipation, and difficulty maintaining direct and conjugate gaze. Her medical history was notable for a cerebrovascular accident 24 years earlier, which resulted in bilateral peripheral visual field deficits and mild memory impairment. She had undergone total knee arthroplasty for severe osteoarthritis and was taking hydrochlorothiazide, atorvastatin, and omeprazole for hypertension, hypercholesterolemia, and gastroesophageal reflux disease, respectively.

Neurologic examination revealed generalized hyperreflexia and spasticity, predominantly in the lower limbs, along with cerebellar dysfunction evidenced by a positive Romberg sign, an ataxic, wide-based gait with impaired tandem walking, and bilateral finger-to-nose dysmetria. Oculomotor assessment revealed impaired smooth pursuit and saccades, with horizontal gaze worsening diplopia. Oral examination demonstrated rhythmic palatal myoclonus.

**Figure 1:**
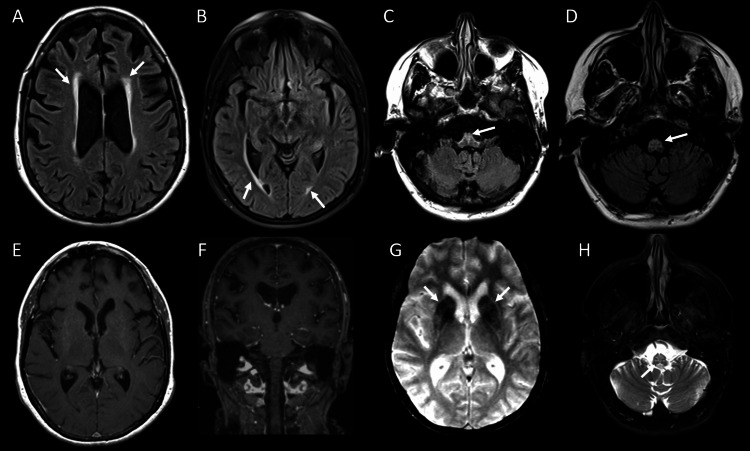
Contrast-enhanced MRI of the brain Brain MRI demonstrates mild to moderate generalized brain volume loss with ex-vacuo prominence of the ventricles and cerebral sulci. On the T2-FLAIR sequence, there is mild diffuse periventricular rim FLAIR hyperintensity (arrows on A and B) without deep white matter involvement. At the level of the medulla and upper cervical spine, there is moderate atrophy and increased T2/FLAIR signal (arrows on C, D, and H) without a mass configuration. Postcontrast imaging (E and F) demonstrates no abnormal enhancement in the basal ganglia, white matter, or brainstem. T2*GRE (G) demonstrates hypointensity, suggesting prominent mineralization in the basal ganglia, particularly in the caudate nucleus and putamen (arrows on G) without atrophy. FLAIR: fluid-attenuated inversion recovery; GRE: gradient recalled echo

Routine laboratory investigations were unremarkable. However, contrast-enhanced MRI of the brain demonstrated a periventricular rim of fluid-attenuated inversion recovery (FLAIR) hyperintensity in the brain (Figure 1).

**Figure 2:**
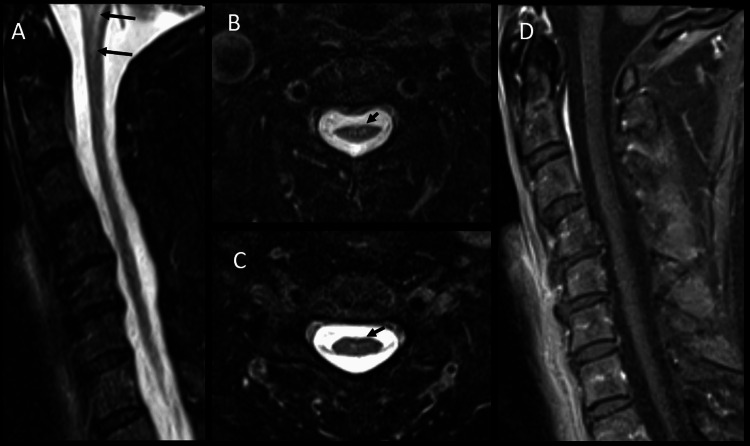
Cervical spine MRI Sagittal T2-STIR (A) and axial T2-STIR at the level of C3 (B) and C5 (C) demonstrate abnormal T2 signal involving the medulla and upper cervical spinal cord at the level of C1 (black arrows on A) and central cord (black arrows on B and C) with mild diffuse cord atrophy. There is no abnormal enhancement within the cord on post-contrast sag T1 fat-saturated imaging (D). STIR: short tau inversion recovery

Additionally, cervical spine MRI showed an abnormal signal and diffuse atrophy involving the cervicomedullary junction and upper cervical spinal cord, characterized by T2/FLAIR hyperintensity in the medulla and central upper cervical cord, along with medullary atrophy (Figure 2).

These clinical and neuroimaging findings, along with a family history of AxD in her 59-year-old brother, prompted expanded genetic testing.

Invitae leukodystrophy panel identified a heterozygous variant in *GFAP*, c.620A>T (p.Glu207Val), located in exon 4. Her brother’s genetic report showed an identical mutation. Additional family history revealed significant neurological issues. While her parents and eight other siblings remained asymptomatic, two sisters (aged 68 and 63 years) exhibited suggestive features such as vocal cord tremors, unsteady gait, and autonomic dysregulation, though without genetic confirmation. Two maternal cousins had genetically confirmed AxD, strongly implicating maternal carriage.

Considering her symptoms, she was initiated on baclofen, tizanidine, and cyclobenzaprine for spasticity and gabapentin for neuropathic discomfort. At the six-month follow-up, she reported modest improvements in tremor control, neuropathic pain, and ambulation, and she continues genetic counseling.

## Discussion

AxD is a rare neurodegenerative disorder characterized by mutations in the *GFAP* gene [1]. Its clinical presentation and genetic diversity, particularly in adult-onset forms, remain an active research area [5]. This case report expands the known genetic spectrum of adult-onset AxD by highlighting a heterozygous missense mutation in *GFAP*. While the identified *GFAP* c.620A>T (p.Glu207Val) variant is absent in major public databases such as ClinVar and gnomAD [6,7], an identical mutation at p.Glu207Val was previously reported in a 52-year-old male with adult-onset AxD [8]. In silico analyses (PolyPhen-2 and MutationTaster) predict this variant to be damaging and disease-causing [9,10]. The glutamate residue at position 207 appears to play a critical role in GFAP function, as alterations at this site have been implicated in disease causation. Multiple missense mutations, including p.Glu207Val (adult onset) [8], p.Glu207Gln (juvenile onset) [11,12], p.Glu207Lys (juvenile onset) [11,12], and splice-site mutations like p.Glu207del (adult onset) [13] and p.Glu207_Lys260del (adult onset) [14] have been associated with AxD (Table 1).

**Table 1:** Review of cases with Glu207 alterations

Case Reports/Studies	Type	Mutation	Exon/Intron	Protein change	Age at onset
van der Knaap et al., 2005 [11]; Li et al., 2005 [12]	missense	c.619G>A	Exon 4	p.Glu207Lys	10 years
van der Knaap et al., 2005 [11]; Li et al., 2005 [12]	missense	c.619G>C	Exon 4	p.Glu207Gln	10 years
Flint et al., 2012 [14]	splice-site	c.619-3C>G	Intron 3	p.Glu207_Lys260del	76 years
Gass et al., 2017 [8]	missense	c.620A > T	Exon 4	p.Glu207Val	52 years
Amano et al., 2021 [13]	splice-site	c.619-1G>A	Intron 3	p.Glu207del	76 years
Current report	missense	c.620A > T	Exon 4	p.Glu207Val	65 years

Unlike the infantile form characterized by rapid neurodevelopmental decline, seizures, and extensive cerebral white matter involvement, adult-onset AxD typically presents in the second to seventh decades with prominent bulbar dysfunction, spasticity, and ataxia, often progressing slowly over the years. Because these features overlap with other adult neurological syndromes, AxD is frequently unrecognized or misdiagnosed without genetic testing or characteristic neuroimaging findings [4,15]. Our patient’s presentation with progressive dysarthria, dysphagia, spastic‐ataxic gait, palatal myoclonus, and oculomotor impairment fits the classic bulbar‐predominant phenotype of AxD. Brain and cervical-spine MRI confirmed the disproportionate medullary and upper cervical cord atrophy with associated T2 signal changes, along with a fine rim of frontal periventricular T2/FLAIR hyperintensity. When coupled with her *GFAP *mutation, this clinicoradiologic fingerprint not only supports the diagnosis and distinguishes AxD from its mimics but also underscores the need for *GFAP* sequencing in adults presenting with unexplained bulbar signs and progressive spasticity.

Although no disease-modifying therapies exist at this point, symptomatic management of spasticity, neuropathic discomfort, and bulbar dysfunction can improve quality of life, and genetic counseling offers at-risk relatives the opportunity for surveillance and early intervention [4]. Emerging approaches that target GFAP expression or aggregation hold promise for future treatment [16].

## Conclusions

The variant's absence in population databases, its prior association with AxD, its predicted deleterious impact, and its familial occurrence alongside a consistent clinical and radiological phenotype strongly suggest its pathogenicity. This case not only emphasizes the pathogenicity of this variant but also supports the autosomal dominant inheritance pattern of the disease. It also highlights the lack of strong genotype-phenotype correlation with variable expressivity, even with the same mutation in the index patient’s family. This phenotypic variability may be due to additional factors affecting disease expression.
